# Gene Replacement and Fluorescent Labeling to Study the Functional Role of Exopolysaccharides in *Bifidobacterium animalis* subsp. *lactis*

**DOI:** 10.3389/fmicb.2017.01405

**Published:** 2017-07-25

**Authors:** Nuria Castro-Bravo, Claudio Hidalgo-Cantabrana, Miguel A. Rodriguez-Carvajal, Patricia Ruas-Madiedo, Abelardo Margolles

**Affiliations:** ^1^Department of Microbiology and Biochemistry of Dairy Products, Instituto de Productos Lácteos de Asturias – Consejo Superior de Investigaciones Científicas Villaviciosa, Spain; ^2^Department of Organic Chemistry, Universidad de Sevilla Sevilla, Spain

**Keywords:** *Bifidobacterium*, exopolysaccharide, gene replacement, fluorescent proteins, NMR, SEC-MALS, biofilms

## Abstract

An extracellular layer of exopolysaccharides (EPS) covers the surface of some *Bifidobacterium animalis* subsp. *lactis* strains, which could be of relevance for its probiotic performance. In order to understand the functional characteristics of *B. animalis* subsp. *lactis*, two isogenic strains that differ in their EPS-producing phenotype, due to a single mutation in the gene Balat_1410, were studied. By means of a double crossover recombination strategy, successfully used for the first time in bifidobacteria, Balat_1410 in the type strain *B. animalis* subsp. *lactis* DSM10140 was replaced by a mutated gene containing a non-synonymous mutation previously associated with the appearance of a mucoid-ropy phenotype. Nuclear magnetic resonance and SEC-MALS analyses showed that the novel strain harboring the mutation acquired a ropy phenotype, due to the production of a high molecular weight (HMW)-EPS that is not produced in the wild-type strain. Fluorescence labeling of both strains with two fluorescent proteins, m-Cherry and Green Fluorescent Protein, was achieved by expressing the corresponding genes under the control of a native selected promoter (the elongation factor Tu promoter). Remarkably, qualitative and quantitative fluorescence analyses demonstrated that the ropy strain displays a lower capability to adhere to human intestinal epithelial cells. In addition, the presence of the HMW-EPS reduced the capability of the producing strain to form biofilms upon three different abiotic surfaces. This work also highlights the fact that different EPS confer variable functional characteristics to the bifidobacterial surface, which may be relevant for the performance of *B. animalis* subsp. *lactis* as a probiotic. The construction of molecular tools allowing the functional characterization of surface structures in next generation probiotics is still a challenging issue that deserves further attention, given the relevant role that such molecules must play in the interaction with the host.

## Introduction

The definition of a probiotic, proposed in 2001 by FAO/WHO, states that is “live microorganisms which when administered in adequate amounts confer a health benefit on the host.” The most commonly commercialized probiotics are some species from *Bifidobacterium* and *Lactobacillus* genera that have been accepted as safe due to their long history of use and they are often delivered into food formulations (Hill et al., 2014). Nowadays, it is becoming more evident that certain intestinal commensal microorganisms could be beneficial to correct microbial dysbioses that have been related with some health disorders; however, they have not been used to promote health yet and, in case they will be applied in this context, they would be treated as novel drugs more than food supplements. These microorganisms can be considered as “next generation probiotics” (NGP) and they are termed as “live biotherapeutic products” (LBP) in the new regulatory framework of the Food and Drug Administration (FDA) of United States of America (O’Toole et al., 2017). Some of the proposed NGP belong to genera *Akkermansia*, *Bacteroides*, and *Faecalibacterium*.

Orally delivered probiotics establish the main contact point with the host at the intestinal mucosa level; in this location, the probiotic-microbiota-cell interplay will drive positive physiological benefits. Despite vast research efforts made into the mechanisms behind the beneficial effects, the manner of probiotic action still remains unclear (Gareau et al., 2010; Wan et al., 2015). The (transitory) contact between bacteria and intestinal epithelial cells might be relevant to initiate this intercellular dialog; the surface microbial associated molecular patterns (MAMPs) interacting with the host pattern recognition receptors (PRR) are involved in triggering the cellular response (Lebeer et al., 2010b; Westermann et al., 2016). One of the most external layers covering the bacterial surface is constituted by exopolysaccharides (EPS), which are carbohydrate polymers whose genetic determinants are present in intestinal bacteria, including most species of the genus *Bifidobacterium* (Ferrario et al., 2016). In fact, some of the beneficial properties attributable to the producing bifidobacteria have been associated with their EPS and their physical-chemical characteristics (Hidalgo-Cantabrana et al., 2014, 2016; Schiavi et al., 2016). Additionally, it has been proven that EPS produced by some NGP, such as *Faecalibacterium prausnitzii*, also has anti-inflammatory properties *in vivo*, thus this bacterium is being proposed as therapeutic agent to treat intestinal inflammatory processes (Rossi et al., 2015).

The development of tools allowing the study of the mechanisms of action, either for well recognized probiotics or NGP, is essential in order to choose those strains which are more valuable for each target population and health benefit. One of the approaches is the use of vectors containing different labeling systems; those applied to lactic acid bacteria and bifidobacteria have recently been reviewed (Landete et al., 2016). A luciferase-based reporter system was developed to monitor the performance of *Bifidobacterium breve* UCC2003 under *in vivo* conditions (Cronin et al., 2008). Different fluorescent proteins have been successfully used as well to label bifidobacteria. In this way, *B. breve*, *B. longum* subsp. *Longum*, and *B. bifidum* were labeled with cyan fluorescent protein (CFP), green fluorescent protein (GFP), yellow fluorescent protein (YFP) or mCherry under the promoter of the *gap* gene (P*_gap_*) of *B. bifidum* (Grimm et al., 2014). Additionally, a GFP fluorescent protein containing a flavin-mono-nucleotide-based cofactor (evoglow-Pp1), which emits fluorescence in the presence/absence of oxygen, was included in a vector under control of the elongation factor Tu (P*_tuf_* from *B. longum*) that replicates in *B. longum* and *B. breve* (Landete et al., 2014). This system, which supposes an advantage to study bifidobacteria in strict anaerobic conditions, was latterly validated under the control of other promoters (Montenegro-Rodríguez et al., 2015).

*Bifidobacterium animalis* subsp. *lactis* is one of the most widely used probiotics and there are several human intervention studies supporting its beneficial effects (Tojo et al., 2014). From an industrial point of view, it is one of the most robust bifidobacterial species which facilitates its inclusion in foods or food supplements (Bogsan et al., 2014). The aims pursued in the current work were: (i) to obtain an EPS-producing variant by means of a double crossover marker-less strategy, which produces a chromosomally stable new variant, (ii) the construction of EPS-producing *B. animalis* subsp. *lactis* strains harboring fluorescent proteins, which have not been reported in literature to date, and (iii) the demonstration that different EPS have an influence on the interaction of the producing strain with biotic and abiotic surfaces. To achieve these goals, a model of *B. animalis* subsp. *lactis* strains, which produced EPS with different physical-chemical characteristics, was initially used. This model was previously developed to demonstrate that a single mutation in the gene Balat_1410, coding for a protein involved in the elongation of the polymer chain, was directly related to a higher abundance of the high molecular weight (HMW)-EPS fraction (about 10^6^ Da) that conferred a ropy-mucoid phenotype to the producing strain (Hidalgo-Cantabrana et al., 2015). Indeed, strains producing HMW-EPS are able to attenuate the immune response (López et al., 2012) and they have been proposed for their application in reducing intestinal inflammatory states (Hidalgo-Cantabrana et al., 2016).

## Materials and Methods

### Bifidobacteria Strains, Plasmids and Culture Conditions

The *B. animalis* subsp. *lactis* and *Escherichia coli* strains, as well as the plasmids and oligonucleotides used in this study, are listed in **Table 1**. *E. coli* DH11S (Invitrogen^TM^, Thermo-Fisher Scientific Inc., Waltham, MA, United States) was grown in Luria-Bertani (LB) broth at 37°C under shaking conditions (200 rpm). Bifidobacterial strains were cultivated in MRSc [MRS (Biokar Diagnostics, Beauvais, Francia) supplemented with 0.25% L-cysteine-HCl (Sigma-Chemical Co., St. Louis, MO, United States)] at 37°C under anaerobic conditions (80% N_2_, 10% CO_2_, 10% H_2_) in a MG500 chamber (Don Whitley Scientific, West Yorkshire, United Kingdom). Bacterial cultures and competent cells were prepared under standardized conditions (Hidalgo-Cantabrana et al., 2015) and ampicillin (100 μg/ml), spectinomycin (100 μg/ml) or erythromycin (2.5 μg/ml) were added when required (**Table 1**).

**Table 1:** Bacterial strains, plasmids, and oligonucleotide primers used in this study.

Strains	Description	Reference
*E. coli* DH11S	*mcrA*Δ(*mrr-hsdRMS-mcrBC*) Δ(*lac-proAB*) Δ(*rec1398*) *deoR rpsL srl-thi*-F’ *proAB*^+^ *lacI*^q^ZΔM15	Invitrogen
*B. animalis* subsp. *lactis*		
DSM10140^T^	Type strain, Plasmid free, EPS^+^, no ropy phenotype	DSMZ collection (yogurt isolated)
IPLA-R1	Plasmid free, EPS^+^, ropy-mucoid phenotype	IPLA collection (bile-salt adapted)
DSM10140-ΔBalat_1410	DSM10140 lacking the gene Balat_1410, no ropy phenotype	Hidalgo-Cantabrana et al., 2015
DSM10140-Balat_1410^S89L^ (=S89L)	DSM10140 mutant obtained after gene integration of Balat_1410^S89L^, EPS^+^, ropy-mucoid phenotype	This work
DSM10140-mCherry	DSM10140 harboring pCAS-mCherry	This work
DSM10140-GFP	DSM10140 harboring pCAS- GFP	This work
S89L-mCherry	DSM10140-Balat_1410^S89L^ harboring pCAS-mCherry	This work
S89L-GFP	DSM10140-Balat_1410^S89L^ harboring pCAS- GFP	This work

**Plasmids**	**Description**	**Reference**

pJL74^a^	*E. coli-Bifidobacterium* cloning vector; Amp^r^ Sp^r^; integrative, non-replicative in *Bifidobacterium*	Hidalgo-Cantabrana et al., 2015
pJL-upst/Balat_1410^S89L^ /dst/ (=pCHC3)	pJL74 containing Balat_1410^S89L^ together with the upstream (3 kb) and downstream (2.7 kb) regions	This work
pAM1^a^	*E. coli-Bifidobacterium* cloning vector; Amp^r^ Em^r^	Alvarez-Martín et al., 2008
pCAS-mCherry	mCherry fluorescent protein gene fused to the elongation factor Tu promoter (P*_tuf_*) of *B. animalis* subsp. *lactis* in pAM1	This work
pCAS- GFP	GFP fluorescent protein gene fused to the elongation factor Tu promoter (P*_tuf_*) of *B. animalis* subsp. *lactis* in pAM1	This work

**Oligonucleotides**	**Sequence (5′–3′)**	**Reference**

Balat_1410 -GR-F^b^	TATATAGGGCCCGTCACCTCGTCACCATGAGC^b^	Hidalgo-Cantabrana et al., 2015
Balat_1410 -GR-R^b^	TATATAAGATCTCCACGAGAGCACACGAAGAC	Hidalgo-Cantabrana et al., 2015
In-Balat_1410 -F	GGTATGATGTGCAGATTCGGCTTC	This work
In-Balat_1410 -R	TACATGGCCGAGAACGAGGTAAACC	This work
Spec-F	GGAGAAGATTCAGCCACTGC	Hidalgo-Cantabrana et al., 2015
Spec-R	TTAGTCGTCGTATCTGAACC	Hidalgo-Cantabrana et al., 2015
P_Tu__F^b^	TATATAAAGCTTACATCCGTTACGAATCACGC	This work
mCh_R^b^	TATATATCTAGATTATTACTTGTACAGCTCGTCC	This work
GFP_R^b^	TATATATCTAGATTATTATTTGTATAGTTCATCC	Thiswork
P_Tu__mCh_F^c^	GTCCAGGAGGACAAAAACAT**ATGGTGAGCAAGGGCGAGG**	This work
mCh_P_Tu__R^c^	**CCTCGCCCTTGCTCACCAT**ATGTTTTTGTCCTCCTGGAC	This work
P_Tu__GFP_F^c^	GTCCAGGAGGACAAAAACAT**ATGCGTAAAGGAGAAGAAC**	This work
GFP_P_Tu__R^c^	**GTTCTTCTCCTTTACGCAT**ATGTTTTTGTCCTCCTGGAC	This work


*^a^Amp^*r*^, Em^*r*^, and Sp^*r*^, resistance to ampicillin, erythromycin, and spectinomycin, respectively. ^b^Restriction enzyme sites are underlined. ^c^Complementary sequences for splicing overlap extension PCR are bold marked.*

### Molecular Techniques

#### Isolation of Chromosomal and Plasmid DNA, and Plasmid Manipulation

Chromosomal DNA from *B. animalis* subsp. *lactis* was isolated using the GeneElute^TM^ Bacterial Genomic DNA kit (Sigma–Aldrich, Dorset, United Kingdom). Plasmid DNA was isolated from *E. coli* using the Qiagen Plasmid Midi kit (Qiagen, Hilden, Germany), whereas the plasmid isolation from recombinant bifidobacteria was performed by means of the GeneElute^TM^ Plasmid Miniprep kit (Sigma–Aldrich). Manufacturer’s recommendations were followed in both cases. For bifidobacterial strains, lysozyme (9 mg/ml, Merck, Darmstadt, Germany) and mutanolysin (5U, Sigma–Aldrich) were added during the lysis step followed by incubation at 37°C for 1 h. DNA concentration was measured in Gene5^TM^ Teck3 Module (BioTek, Vermont, United States).

For plasmid constructions, PCRs were performed using Platinum^®^ Pfx DNA Polymerase (Invitrogen^TM^). Digestions and ligations were made with restriction endonucleases from Takara (Takara Bio Group, Otsu, Japan) and with T4 DNA ligase from Invitrogen^TM^, respectively. All reagents were used according to the manufacturers’ instructions. PCR products were checked by electrophoresis in TAE buffer [40 mM TRIS, 20 mM acetic acid, 1 mM EDTA (pH 8)] on 1% agarose gels and then stained with ethidium bromide (0.5 μg/ml). DNA purification from the agarose gels was performed using QIAquick Gel Extraction Kit (QIAgene) and sequenced at Macrogen Inc. (Seoul, South Korea). BLAST algorithm was used for sequence similarity analysis. Finally, Eurx-Taq DNA Polymerase from Roboklon GmbH (Berlin, Germany) was used to check plasmid constructions in *E. coli* and plasmid integration in the bifidobacterial chromosome.

#### Gene Replacement: Plasmid Construction and Double Crossover Events

The chromosomal DNA from *B. animalis* subsp. *lactis* IPLA-R1 was used as a template for PCR amplification using the specific primers Balat_1410-GR-F/R (**Table 1**) for the construction of the plasmid for gene replacement. These primers amplify 7.1 kb which contain the Balat_1410^S89L^ gene (Hidalgo-Cantabrana et al., 2015) and its flanking regions: upstream (3 kb) and downstream (2.7 kb). The PCR product was digested with *Apa*I and *Bgl*II and cloned into pJL74 previously digested with the same enzymes. Ligation was performed overnight at 16°C and the ligation mixture was purified and transformed into *E. coli* DH11S electrocompetent cells. The resulting plasmid was named pCHC3 (pJL-upst/Balat_1410^S89L^/dst) and was introduced into *B. animalis* subsp. *lactis* DSM10140-ΔBalat_1410 electrocompetent cells prepared as previously reported (Hidalgo-Cantabrana et al., 2015). After transformation, bifidobacterial cells were immediately recovered in 2 ml of MRSc and incubated at 37°C under anaerobic conditions for 4–6 h before plating onto the same agar-medium containing spectinomycin (100 μg/ml). Plates were then incubated for 48 to 72 h at 37°C in anaerobic conditions. Transformants were checked for plasmid integration into the chromosome (single crossover) by PCR using the specific primers In-Balat_1410-F/R and Spec-F/R (**Table 1**). At this point of the experiment, two of the checked colonies acquired the visually recognizable ropy phenotype (having the integrated plasmid) and they were selected to be grown in 10 ml MRSc without antibiotic. Two subcultures (per day) were made for 5 days to force the loss of the plasmid (second crossover) and, afterward, these bacterial cultures were plated onto agar-MRSc without antibiotics for 48 h. Several colonies were picked up and each of them was grown in agar-MRSc, with and without spectinomycin, to select the non-antibiotic resistant colonies due to the loss of the plasmid. These colonies were checked by PCR using the specific primers In-Balat_1410-F/R and Spec-F/R to analyze the presence of Balat_1410^S89L^ and the absence of spectynomicin-resistance genes into the chromosome; besides, the ropy phenotype, associated with the presence of Balat_1410^S89L^, was useful for the selection of the right colonies. Thus, one strain with a ropy character, then putatively carrying the Balat_1410^S89L^ gene, and being sensitive to spectynomicin, was selected and named DSM10140-Balat_1410^S89L^, or S89L in its abbreviated form (**Table 1**). To confirm its genetic background, the chromosomal DNA from S89L was obtained and an inner fragment (1 kb) of Balat_1410^S89L^ gene, containing the mutation (C to T transition) responsible for the ropy trait (Hidalgo-Cantabrana et al., 2015), was amplified using the In-Balat_1410-F/R primers. The genetic background of the *eps* cluster surrounding the insertion and the complete insert was also checked with a set of primers that amplify 1 kb overlapping fragments (data not shown). All these PCR products were sequenced at Macrogen Inc. to confirm the absence of undesirable mutations.

#### Strain Labeling Using Fluorescence Plasmids

Plasmids harboring fluorescent proteins under the control of the elongation factor Tu promoter from *B. animalis* subsp. *lactis* were constructed using splicing overlap extension PCR strategy to fuse the DNA sequences (Vallejo et al., 1994). The “elongation factor Tu” promoter was amplified from the chromosomal DNA of DSM10140 strain using the specific primers P_Tu__F/mCh_P_Tu__R (**Table 1**). The gene encoding mCherry fluorescent protein was amplified from the pVG-mCherry plasmid (Grimm et al., 2014) with specific primers P_Tu__mCh_F/mCh_R. The PCR products that have complementary tails, between each other, were size-checked in 1% agarose gel. Then, splicing overlap extension PCR was performed to fuse both fragments. The same protocol was followed to fuse the elongation factor Tu promoter to GFP gene: the Tu promoter was amplified using specific primers P_Tu__F/GFP_P_Tu__R, and GFP gene was obtained from the pVG-GFP plasmid (Grimm et al., 2014) using specific primers P_Tu__GFP_F/GFP_R. Fused DNA fragments were checked by electrophoresis and purified from agarose gels. The fused DNA fragments were digested with *Hind*III and *Xba*I at 37°C for 3 h, as well as the plasmid pAM1 (Alvarez-Martín et al., 2008) which was also dephosphorylated. Digestions were also purified from agarose gels and used to perform overnight ligations at 4°C. Electrocompetent *E. coli* DH11S cells were transformed with the ligation mixtures and selection of clones was performed by adding ampicillin (100 μg/ml) to the culture medium. The resulting plasmids were named pCAS-mCherry and pCAS-GFP (**Table 1**). The strains *B. animalis* subsp. *lactis* DSM10140 and S89L were transformed with these plasmids and four fluorescent clones were selected by adding erythromycin (2.5 μg/ml) to the culture medium; the recombinant strains were named as DSM10140-mCherry, DSM10140-GFP, S89L-mCherry and S89L-GFP (**Table 1**).

### Qualitative and Quantitative Fluorescence Detection

#### Fluorescence Scanning

The fluorescent bifidobacterial strains were grown onto the surface of agar-MRSc containing erythromycin, for 3 days at 37°C under anaerobic conditions. The fluorescence of the colonies was checked in the Typhoon 9400 scanner (GE Healthcare, Biosciences, Uppsala, Sweden). GFP was excited with blue laser (488 nm) and emission was detected with 526 nm band-pass filter. In the case of mCherry, excitation was performed with red laser (633 nm) and emission was acquired with 580 nm band-pass filter. These plates were scanned at a resolution of 100 μm pixel size.

#### Fluorescence Microscopy and Confocal Scanning Laser Microscopy (CSLM)

To visualize the bifidobacteria expressing the fluorescent proteins, overnight grown cultures (in MRSc + erythromycin) were washed, placed on a slide covered with coverslip No.1 (0.13–0.16 mm thick). These preparations were observed with the Leica DMi8 inverted microscope (Leica Microsystems GmbH, Heidelberg, Germany), using a 100× oil immersion objective. The FITC filter cube (excitation 480/40, emission 527/30) and RHOD filter cube (excitation 546/10, emission 585/40) were used for visualization of the bifidobacteria harboring GFP or mCherry proteins, respectively.

Fluorescent (mCherry) bifidobacteria adhered on the top of the intestinal cell line HT29 or into glass slides (as will be described next) were visualized with the Leica TCS AOBS SP8 X confocal inverted microscope [External Service Unit (ESU) of the University of Oviedo, Asturias, Spain]. To visualize DAPI fluorochrom, samples were excited at 405 nm, by a blue-violet laser diode, whereas to detect the mCherry they were excited at 587 nm by a white light laser. Z-stacks of HT29 monolayers or bifidobacterial biofilms upon glass μ-slides were acquired with a 63×/1.4 oil objective. When needed, a 2.50 optical zoom was used to acquire images of detailed regions. Image captures were reordered and processed with the Leica Application Suit X software (version 1.8.1.13759, Leica).

#### Fluorescence Spectrometry

Fluorescence quantification was performed with overnight cultures of the four fluorescent bifidobacteria, as well as the two parental DSM10140 and S89L strains used as negative controls. Cells were washed with PBS and standardized to an equal OD_600 nm_; additionally, they were plated in the corresponding (with and without antibiotic) agar-MRSc media. Afterward, the standardized bacterial suspensions were 10-fold concentrated and from them, serial (half) dilutions were prepared in PBS. 96-well Lumitrack^TM^ 600 white polystyrene plates (VWR, Radnor, PA, United States) were filled with 200 μl (per well) of each bifidobacterial dilution. Fluorescence was measured on the Cary Eclipse (Varian Ibérica, S.A. Madrid, Spain) fluorescence spectrometer using the following conditions: 470 nm excitation/525 nm emission, for GFP quantification, and 585 nm excitation/610 nm emission for mCherry quantification. The corresponding fluorescence background, determined from the control samples (parental, non-labeled bifidobacterial suspensions), was subtracted from data obtained for the fluorescent strains. This experiment was performed with three biological replicates. Finally, linear regression equations between the fluorescence emitted and the number of bacteria (Log CFU/ml) were calculated, as well as the corresponding coefficient of determination (*R*^2^) that shows how well the data fits to the linear regression.

#### Flow Cytometry

Fluorescence of bifidobacterial suspensions, obtained as previously described, were also quantified in the Cytomics FC500 (Beckman Coulter, Barcelona, Spain) located in the ESU from the University of Oviedo. A fix acquisition time of 90 s with “hi” acquisition speed was used. The 488 nm laser was applied for the excitation of both fluorochromes and the selection of the bifidobacterial population was made by means of FSC log/SSC log (size/complexity). This gate was used to plot the FL1 (for GFP detection) vs. FL3 (for mCherry detection) histograms; the filters 525/40 and 620/30 were used for the detectors FL1 and FL3, respectively. The absence of auto-fluorescence was checked in the corresponding non-labeled bacteria (Supplementary Figure S1). In the labeled strains the recorded fluorescence was compensated to avoid the overlapping of both fluorochromes. Finally, serial dilutions of the samples (from 1/2 to 1/100) were measured and the linear regression equations between the “fluorescence emitted” (total number of events multiplied by the mean fluorescence intensity) and the number of bacteria (CFU/ml); the *R*^2^ coefficients were calculated as well (Supplementary Figure S2).

### Chemical Analysis of Purified EPS

#### EPS Purification

The EPS from strains DSM10140, S89L and IPLA-R1 were isolated from the bifidobacterial biomass collected with water from the surface of agar-MRSc plates as previously described (Ruas-Madiedo et al., 2010). Each bacterial suspension was mixed with 1 volume of 2 M NaOH and kept overnight at room temperature under mild shaking. Bacteria were eliminated by centrifugation and the EPS from the supernatant was precipitated with two volumes of chilled absolute ethanol for 48 h at 4°C. Precipitated sediment was collected with ultra-pure water and dialyzed against water, using dialysis tubes of 12–14 kDa molecular mass cut off (Sigma), at 4°C for 3 days with a daily change of water. Finally, each dialyzed sample was freeze-dried in order to obtain the crude-EPS material from each strain. To isolate the HMW EPS-fraction from strains S89L and IPLA-R1, the crude-EPS (25 mg) was dissolved in ultra-pure water (10 ml), dialysed (against water, for 72 h at 4°C) using Spectra/Por Float-A-Lyser 100 kDa MWCO tubes (Sigma), and the content of these dialyzed tubes was freeze-dried (Leivers et al., 2011).

#### SEC-MALLS Analysis

The molar mass distribution of the crude-EPS and HMW-EPS, as well the quantification of the relative amount of the different size-fractions, were performed by means of size exclusion chromatography (SEC); a chromatographic system (Waters, Milford, MA, United States) coupled in series with a refractive index (RI) detector (Waters) and with a multi-angle laser light scattering detection (MALLS, Dawn Heleos II, Wyatt Europe GmbH, Dembach, Germany) was used as previously described (Nikolic et al., 2012).

#### NMR Analysis

The HMW-EPS fractions were analyzed by nuclear magnetic resonance (NMR) at the facilities of the University of Seville (Seville, Spain). A sample of 10 mg was deuterium-exchanged several times by freeze-drying from D_2_O and then examined in solution (10 mg/750 mL of 99.96% D_2_O, Sigma–Aldrich). Spectra were recorded on a Bruker AV500 spectrometer (Bruker BioSciences, Madrid, Spain) operating at 500.13 MHz (^1^H). Chemical shifts were given in ppm, using the HDO signal (4.31 ppm at 343 K) as reference (Gottlieb et al., 1997). The 2D heteronuclear one-bond proton-carbon correlation experiment was registered in the ^1^H-detection mode via single-quantum coherence (HSQC). A data matrix of 256 × 1K points was used to digitize a spectral width of 5208 in F2 and 22522 Hz in F1. ^13^C decoupling was achieved by the GARP scheme. Squared-cosine-bell functions were applied in both dimensions, and zero-filling was used to expand the data to 1K × 1K.

### Adhesion to HT29

The intestinal epithelial cell line HT29 (ECACC 91072201, European Collection of Cell Cultures, Salisbury, United Kingdom) was used to test the adhesion capability of the fluorescence-labeled and non-labeled bifidobacterial strains; *B. animalis* subsp *lactis* BB-12 was used as reference strain. HT29 was maintained under standard conditions using McCoy’s medium (MM, Sigma) supplemented with 10% fetal bovine serum (Sigma) and with a mixture of antibiotics (50 μg/ml penicillin, 50 μg/ml streptomycin, 50 μg/ml gentamicin and 1.25 μg/ml amphotericin B, Sigma). The adhesion experiments were performed upon 11-day old HT29 monolayers grown in microtiter plates. Bifidobacterial suspensions, prepared in MM (without antibiotics), were added to each well at ratio 10:1 (bifidobacteria: HT29) and incubated for 1 h at 37°C/5% CO_2_ (Nikolic et al., 2012). For the non-labeled strain the number of bacteria added and bacteria adhered was determined by plating on agar-MRSc and the adhesion percentage was calculated as the ratio between the bacteria adhered with respect to the bacteria added (Nikolic et al., 2012). For the fluorescence-labeled bacteria, the fluorescence was measured by means of flow cytometry, as previously described, and values of absolute fluorescence were used to present the adhesion results. In addition, experiments of competition between the “fluorescence-labeled, ropy” strain and the “non-labeled, non-ropy” strain (or “fluorescence-labeled, non-ropy” vs. “non-labeled, ropy”) for adhesion to HT29 were carried out; in this case, both bacteria were added in equal amounts to the cell line (10:1, ratio bacteria: HT29) and the absolute fluorescence was measured.

### Bifidobacterial Biofilm Formation upon Abiotic Surfaces

The capability of the ropy and non-ropy EPS-producing bifidobacterial strains to form biofilms was determined using different procedures and abiotic surfaces. A method, based on impedance measurement, recently described by Gutiérrez et al. (2016) to monitor in real time the formation of bacterial biofilms was used. In short, the real time cell analyzer (RTCA) equipment xCelligence RTCA-DP (ACEA Bioscience Inc., San Diego, CA, United States) was introduced in an incubator, at 37°C with 5% CO_2_, at least 2 h before the experiments. Bifidobacterial cultures were washed twice with PBS to prepare standardized suspensions in fresh MRSc (∼10^9^ cfu/ml) which were placed in the wells (100 μl/well) of specific E-plates (ACEA Bioscience Inc.) coated with gold-microelectrodes that are able to transmit the impedance signal. The RTCA software 2.0 (ACEA Bioscience) was used for data collection and the biofilm formation was followed for 46 h, using three biological replicates for each strain; finally, the wells were stained with crystal violet, as will be described next. Biofilms were also formed upon polystyrene plates (96-well microplates Nunc, Thermo-Fisher Scientific Inc.), upon microscope cover glasses (No. 1, 18 mm diameter, Marienfeld GmbH, Lauda-Königshofen, Germany) previously sterilized by autoclaving (121°C, 20 min) which were placed into 6-well microplates (Thermo Fisher Scientific Inc.), and upon the surface of μ-slide-2-well glass bottom (Ibidi GmbH, Martinsried, Germany). After 24-h incubation at 37°C in anaerobic chamber, these biofilms were also stained with crystal violet. Additionally, the fluorescence-labeled bifidobacterial biofilms (incubated in darkness) formed upon cover glasses were visualized under the epifluorescence microscope and those formed upon the surface of μ-slide-2-well glass bottom were detected with the CSLM.

#### Crystal Violet Staining

The end-point crystal violet method was used to quantify the bifidobacterial biofilm formation upon the three abiotic surfaces used (Gutiérrez et al., 2016). In brief, supernatants from different abiotic-material wells were removed and biofilms washed twice with PBS, dried for 15 min at room temperature and stained with a solution (0.1% w/v) of crystal violet for 15 min. Then, biofilms were gently washed with water, de-stained with a solution (33%) of acetic acid for at least 15 min and, finally, the absorbance of the supernatants was measured at 595 nm in a Microplate Benchmark Plus (Bio-Rad, Hercules, CA, United States) spectrophotometer.

### Statistical Analysis

The statistical package IBM SPSS Statistics for Windows Version 22.0 (IBM Corp., Armonk, NY, United States) was used to assess differences among strains by means of one-way ANOVA followed, when needed, by SNK (Student-Newman–Keuls, *p* < 0.05) mean comparison test. The legend of each figure indicates the analysis performed. Finally, the *R*^2^ coefficients, that reflect the adjustment to linear regression equations between different parameters, were calculated.

## Results and Discussion

### Generation of a Ropy Strain with a Non-synonymous Mutation in the Gene Balat_1410

In a previous work, an isogenic mutant derived from the type strain *B. animalis* subsp. *lactis* DSM10140 by removing the gene Balat_1410, using a knockout mutation system based on the integrative plasmid pJL74, was constructed (Hidalgo-Cantabrana et al., 2015). Our first aim in the present study was to reintroduce into the genome of *B. animalis* subsp. *lactis* DSM10140-ΔBalat_1410 a mutated Balat_1410 gene containing a non-synonymous, single nucleotide mutation previously associated with the appearance of a mucoid-ropy phenotype in the strain DSM10140-ΔBalat_1410-pAM1-Balat_1410^S89L^ (Hidalgo-Cantabrana et al., 2015). To do that, a double-crossover marker-less strategy previously used for the deletion of the gene, was followed. The strain DSM10140-ΔBalat_1410 was transformed with the plasmid pCHC3 (**Table 1**) containing a fragment of approximately 7 kb amplified from the genome of the strain IPLA-R1 (**Table 1**), that includes the regions immediately located upstream and downstream of the gene Balat_1410 as well as the mutated gene Balat_1410^S89L^ between those regions. Gene integration was achieved as previously described (Hidalgo-Cantabrana et al., 2015), resulting in *B. animalis* subsp. *lactis* DSM10140-Balat_1410^S89L^ (abbreviated as S89L), a strain that has exactly the same genetic background as *B. animalis* subsp. *lactis* DSM10140 which underwent a gene replacement that resulted in a C to T transition in the gene Balat_1410 at position 266, causing a codon change in position 89 (a serine is substituted by a leucine). Although there are several works that have reported gene deletion and interruption systems in bifidobacteria, including *B. breve* (Ruiz et al., 2012), *B. longum* (Fukuda et al., 2011; Hirayama et al., 2012) and *B. animalis* subsp. *lactis* (Arigoni and Delley, 2008; Hidalgo-Cantabrana et al., 2015), to our knowledge this is the first report of a successful gene replacement strategy in bifidobacteria. Due to the lack of genetic tools to introduce specific point mutations in bifidobacterial genomes, our methodology represents a suitable alternative to overcome this limitation.

### Analysis of EPS Synthesized by the Recombinant Ropy Strain

**FIGURE 1:**
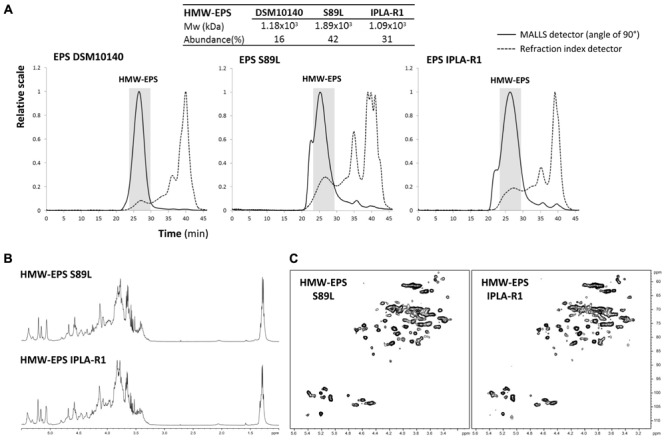
Physical-chemical analysis of EPS isolated from three *B. animalis* subsp. *lactis* strains. SEC-MALLS analysis of the EPS-DSM10140, EPS-S89L and EPS-IPLA-R1 showing the molecular weight (Mw) and relative abundance of the high molecular weight (HMW) fraction in each polymer **(A)**. ^1^H-NMR (500 MHz, 343 K) spectra **(B)** and 500-MHz^1^H-^13^C- HSQC spectra **(C)** of HMW-EPS purified from EPS-IPLA-R1 and EPS-S89L polymers.

In the EPS synthesized by the two ropy strains *B. animalis* subsp. *lactis* IPLA-R1 (parental) and S89L (recombinant) the HMW-EPS fraction (about 1 × 10^6^ Da) was present in a higher proportion than in the non-ropy DSM10140 (parental) strain (**Figure 1A**). It should be noticed that the polymer material purified from the three strains, with the procedures used in this study, is the cell-associated EPS but not that liberated into the medium. Previously, the production of the HMW-EPS was correlated with the occurrence of the mucoid-ropy appearance in a recombinant strain harboring the mutated gene in a multi-copy plasmid (Hidalgo-Cantabrana et al., 2015); thus, currently this finding was reinforced with the acquisition of the ropy phenotype in the novel S89L having the single mutation stabilized into the chromosome. After purification of the HMW-EPS fraction from polymers synthesized by IPLA-R1 and S89L strains, one- and two-dimensional NMR analyses revealed an identical chemical composition (**Figures 1B,C**). These physical-chemical analyses undoubtedly prove that the gene replacement strategy applied to obtain the recombinant S89L strain was successful to introduce the mutation linked to the production of the HMW-EPS. The structural repeating unit of the HMW-EPS was already described for strain IPLA-R1 (Leivers et al., 2011); it is an hexapolysaccharide with 50% rhamnose content, and it is very similar to that reported for strain *B. animalis* subsp. *lactis* LKM512 (Uemura and Matsumoto, 2014).

### Expression of Fluorescent Proteins in *B. animalis* subsp. *lactis* and Fluorescence Detection

**FIGURE 2:**
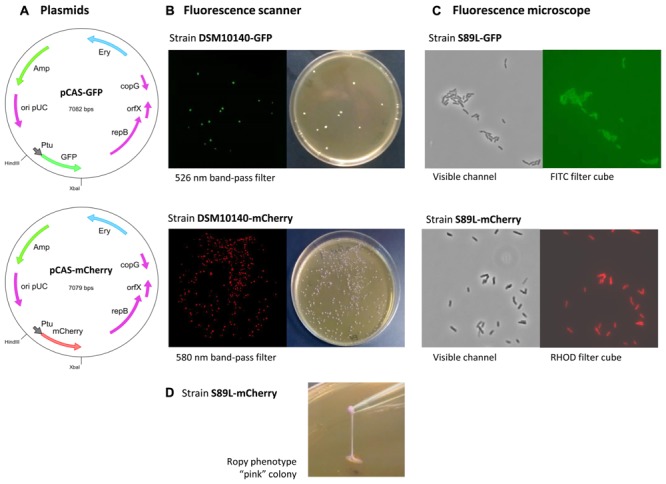
Physical map of pCAS-GFP and pCAS-mCherry, in which the GFP and mCherry genes are located downstream from the elongation factor Tu promoter (P*_tuf_*) from *B. animalis* subsp. *lactis* DSM10140 **(A)**. Detection of fluorescence green (top) or red (bottom) colonies of *B. animalis* subsp. *lactis* DSM10140-GFP and DSM10140-mCherry, respectively, using the Typhoon 9400 scanner fluorescence scanner (left hand photographs) and aspect of the colonies visualized with a conventional camera (right hand photographs) **(B)**. Fluorescent *B. animalis* subsp. *lactis* S89L, transformed with pCAS-GFP (top) and pCAS-mCherry (bottom), visualized with the Leica DMi8 inverted microscope using a 100× oil immersion objective **(C)**. Strain *B. animalis* subsp. *lactis* S89L-mCherry showing a colored pink colony with a ropy phenotype denoted for the formation of a long, unbreakable filament **(D)**.

**FIGURE 3:**
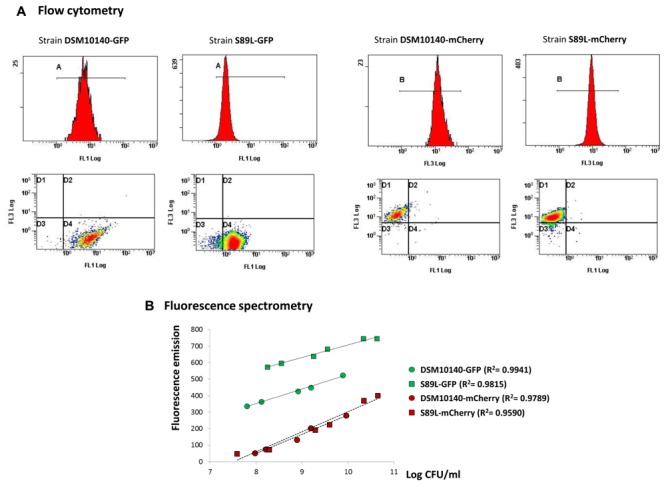
Fluorescence quantification of GFP (the two left panels) and mCherry proteins (the two right panels) of the bifidobacterial strains harboring the corresponding plasmids using the Cytomics FC500 flow cytometer; see Supplementary Figure S2 for correlation with bacterial enumeration in agar-MRSc **(A)**. Linear regression between the fluorescence emitted, recorded by the CaryEclipse fluorescence spectrometer, and the bacterial counts (Log CFU/ml) of seriated dilutions of the fluorescent bifidobacterial suspensions; the coefficients of determination (*R*^2^), showing how well data fit to the regression line equations, are indicated in bold letters **(B)**.

*Bifidobacterium animalis* subsp. *lactis* DSM10140 and S89L were transformed with the plasmids pVG-GFP and pVG-mCherry, containing the genes coding for the proteins GFP and mCherry, respectively. It was reported that these plasmids were successfully used for the fluorescent detection of *B. longum*, *B. breve*, and *B. bifidum* strains, grown in similar conditions to those used in our study (Grimm et al., 2014). However, no fluorescence was detected in our *B. animalis* subsp. *lactis* strains using fluorescence spectrometry techniques, suggesting that either the genes are not expressed or the proteins do not emit fluorescence under our experimental conditions. Since gene expression in the plasmids pVG-GFP and pVG-mCherry is under the control of the promoter of the glyceraldehyde-3-phosphate dehydrogenase gene of *B. bifidum* (P*_gap_*), a specific promoter of *B. animalis* subsp. *lactis* was investigated which could be suitable to trigger the expression of the GFP and mCherry genes in our strains. Our previous work on bifidobacteria allowed us to depict a detailed protein map of the most abundant cytoplasmic proteins in their soluble proteome (Sánchez et al., 2005, 2007). Indeed, one of the most abundant proteins in the soluble proteome of some bifidobacteria is the elongation factor tu (the product of the *tuf* gene; Sánchez et al., 2005; Wei et al., 2016). Furthermore, previous reports have shown that the P*_tuf_* of *B. longum* is a suitable strong and constitutive promoter able to trigger the expression of fluorescent proteins in *B. longum* and *B. breve* (Landete et al., 2014). These previous findings indicate that the *tuf* promoter could be a good candidate for the expression of heterologous genes in *B. animalis* subsp. *lactis*. With this in mind, the promoter of the glyceraldehyde-3-phosphate dehydrogenase, originally present in the plasmids pVG-GFP and pVG-mCherry, was replaced by the upstream region of the *tuf* gene of *B. animalis* subsp. *lactis* DSM10140, containing P*_tuf_*, yielding the plasmids pCAS-mCherry and pCAS-GFP (**Table 1** and **Figure 2A**). These plasmids were used to transform *B. animalis* subsp. *lactis* DSM10140 and *B. animalis* subsp. *lactis* S89L; green and red fluorescence in the resulting strains (DSM10140-mCherry, DSM10140-GFP, S89L-mCherry and S89L-GFP) were detected by fluorescence scanning (**Figure 2B**) and fluorescence microscopy (**Figure 2C**). The ropy phenotype, denoted by the formation of a filament, was detected in the strain S89L-mCherrey which also acquired a pink color in the colony (**Figure 2D**). Furthermore, a quantitative analysis of the fluorescently labeled bifidobacteria was performed using flow cytometry (**Figure 3A**), and the fluorescence signal was correlated with bacterial counts by a linear regression analysis (Supplementary Figure S2). Our results showed that the fluorescence quantification of the strains labeled with the two fluorescent proteins correlate with the bacterial counts in agar plates. The coefficients of determination (*R*^2^) obtained in these analysis were in all cases higher than 0.95, indicating the suitability of our approach to quantify fluorescently labeled bifidobacterial populations in a range of 2 log count units (**Figure 3A** and Supplementary Figure S2). Very good fits to the linear regression were also obtained in a range of 3 log count units when the fluorescence quantification was carried out using spectrometric techniques (**Figure 3B**). Previous works have shown that other species, such as *B. longum*, *B. bifidum*, and *B. breve*, can be fluorescently labeled with a variety of proteins, including GFP, m-Cherry, Yellow Fluorescent proteins and Cyan Fluorescent proteins (Grimm et al., 2014; Landete et al., 2014). However, it is worth highlighting that, to the best of our knowledge, this is the first report describing the fluorescent labeling of *B. animalis* subsp. *lactis*, which opens new possibilities to track the functional properties of this *Bifidobacterium* under *in vitro* and *in vivo* conditions.

Regarding the stability of the fluorescence emission of GFP and mCherry, the fluorescence signal of GFP was quite unstable compared with that of m-Cherry. Once the cell growth was stopped, the cells were not able to emit fluorescence for longer than 1 h, independently of the technique used to measure the GFP fluorescence. A quick decrease in the fluorescence in the two *B. animalis* subsp. *lactis* strains analyzed was observed. However, m-Cherry fluorescence was more intense and stable for longer periods of time (a significant decrease of the fluorescent signal after 24 h at RT was not observed) (data not shown). Therefore, the strains labeled with m-Cherry were selected to perform the experiments described in the following sections of this work.

### Application of Fluorescence Labeling to Assess the Role of Ropy EPS in the Adhesion to HT29

**FIGURE 4:**
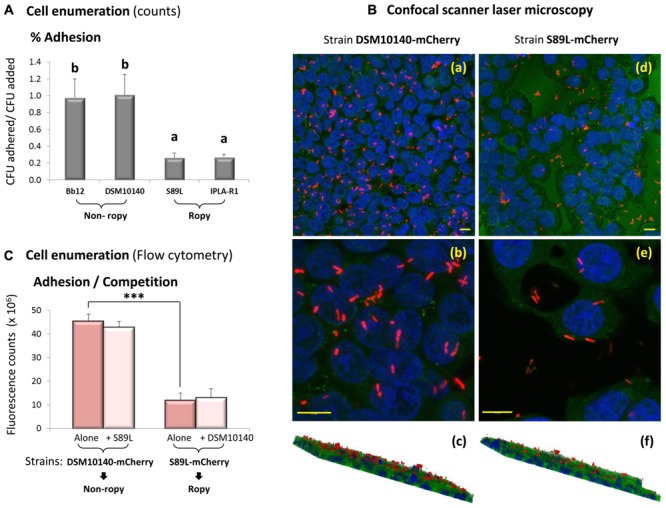
Adhesion to the intestinal cell line HT29 of four *B. animalis* subsp. *lactis* strains: DSM10140 and Bb12 display a non-ropy phenotype and S89L and IPLA-R1 display a ropy phenotype. Bars that do not share a common letter are statistically (*p* < 0.05) different **(A)**. Visualization of DSM10140-mCherry (a, b, and c) and S89L-mCherry (d, e, and f) strains adhered to HT29 using the TCS SPE confocal module of the Leica DMi8 inverted microscope (bars 10 μm). The nucleus was DAPI-stained in blue (excited at 405 nm), the green color corresponds with the auto-fluorescence emitted by cytoplasmic molecules of the cell line, and the fluorescent bifidobacteria were detected in red (excited at 561 nm). The bottom photographs (c and f) represent a rotated 3D-image obtained from the Z- projection (10-XY slides, thickness about 13–15 μm) showing the integrity of the HT29 monolayer covered by a “grass” of fluorescent *B. animalis* subsp. *lactis* strains **(B)**. Adhesion to HT29 of strains DSM10140-mCherry and S89L-mCherry (red bars) quantified by means of flow cytometry; statistical difference between both strains are indicated with asterisks (*p* < 0.01). The pink bars show the adhesion of the fluorescent strains in competition with the non-fluorescent counterpart strains **(C)**.

In the last consensus statement in the probiotics field revised in 2014, it was considered that the FAO/WHO Guidelines proposed in 2002 (Food, and Agricultural Organization of the United Nations, and World Health Organization [FAO/WHO], 2002) still provide a useful approach to search for and validate probiotic candidates (Hill et al., 2014). Among the *in vitro* tests carried out to study new strains, the capability to adhere to mucus and/or intestinal epithelial cells is extensively analyzed before undertaking *in vivo* studies. In this study, conventional (culturing) techniques have been used to test the adhesion to the human intestinal cell line HT29 of the recombinant *B. animalis* subsp. *lactis* S89L strain in comparison with their isogenic parental strains. The ropy S89L and IPLA-R1 strains, both synthesizing in higher abundance the HMW-EPS, adhered significantly less to HT29 than DSM10140 and Bb12 (a probiotic reference) strains (**Figure 4A**). This result was contrary to that obtained in a previous work with the recombinant (ropy) DSM10140-ΔBalat_1410-pAM1-Balat_1410^S89L^ strain harboring the mutated Balat_1410 gene in the multicopy plasmid pAM1 (Hidalgo-Cantabrana et al., 2015). In fact, the three recombinant strains carrying pAM1 plasmid showed higher adhesion values (above 3%) to HT29 than the values obtained in the current work for the better adherent (around 1%) strains (DSM10140 and Bb12). Thus, in order to clarify this, apparently, contradictory result, the fluorescently labeled strains were used to address their capability to adhere to HT29 by means of different techniques and using the new experimental models in which there is a single copy of Balat_1410 (or Balat_1410^S89L^) in the chromosome. The CSLM visualization corroborated that the ropy, HMW-EPS producing S89L-mCherry strain adhered to HT29 in lower proportions than the non-ropy DSM10140-mCherry strain (**Figure 4B**). Similarly, the quantitative flow cytometry technique showed about a fivefold reduction (*p* < 0.05) in the adhesion of the first strain with respect to the second (**Figure 4C**). Additionally, the simultaneous addition of a fluorescent strain in combination with its non-labeled counterpart (e.g., strain DSM10140-mCherry and S89L) did not modify the adhesion capability of both fluorescent ropy and non-ropy strains (**Figure 4C**); this indicates that the higher abundance of the HMW-EPS did not represent a disadvantage for *in vitro* competition to adhere to the epithelial cell line. Thus results obtained in the current study support the generalized finding that EPS of high molecular mass make difficult the adhesion of the producing strain to the intestinal epithelium because they could hinder the accessibility of other molecules acting as adhesins (Lebeer et al., 2009; Nikolic et al., 2012; Horn et al., 2014). However, the role of bacterial EPS on the adhesion capability of the producing strain has not been clearly established since it is highly dependent on the producing strain, the intrinsic characteristics of the polymer, as well as on the biological model of study used. As an illustrative example, *in vitro* studies showed that the EPS surrounding *Lactobacillus rhamnosus* GG reduced the adhesion of the strain to the intestinal cell line Caco2 (Lebeer et al., 2009) because the accessibility of the pili acting as strong adhesin to enterocytes was hindered (Lebeer et al., 2012). However, *in vivo* studies showed that this EPS forms a protective shield against host-antimicrobial secreted factors which helps the persistence of strain GG in the gut (Lebeer et al., 2010a). Thus, production of EPS by *L. rhamnosus* GG is relevant to keep an optimal performance, i.e., a balance between protection and adhesion.

### Role of Ropy EPS in the Biofilm Formation upon Abiotic Surfaces

**FIGURE 5:**
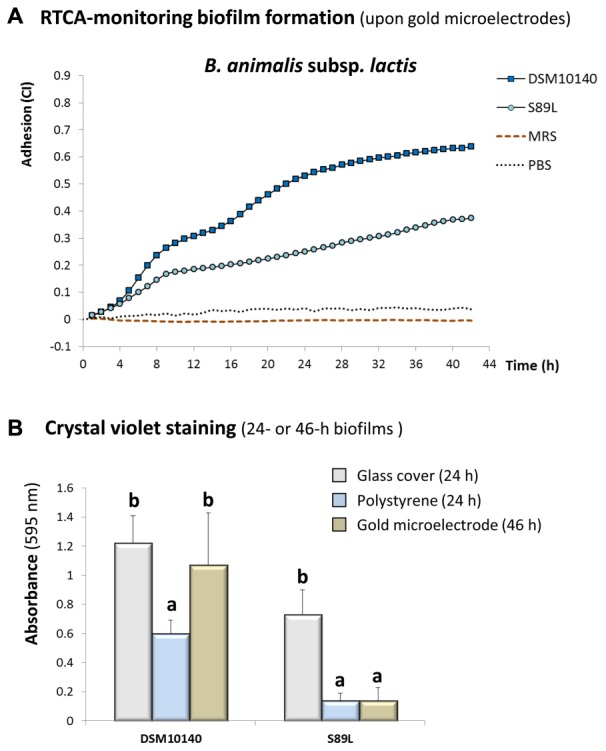
Monitoring in real time the formation of biofilms by strains *B. animalis* subsp. *lactis* DSM10140 and S89L upon (gold-microelectrodes) E-plates in the RTCA (real time cell analyzed) equipment **(A)**. Biofilms formed by the same strains in three abiotic surfaces and stained, at different end-point times, with the crystal violet method; within each strain, bars that do not share a common letter are significantly (*p* < 0.05) different **(B)**.

Another important role that EPS synthesized by probiotic bacteria could play in the gut ecosystem is the capability of reducing the activity of pathogens in the intestine, including their adhesion to the intestinal epithelium. This has been proved with *in vitro* (Ksonzeková et al., 2016; Zivkovic et al., 2016) as well as *in vivo* studies (Fanning et al., 2012; Chen et al., 2014; Nácher-Vázquez et al., 2015) using different intestinal cell lines and animal models, respectively. It was postulated that the mechanism behind this EPS protection could be the formation of a protective “biofilm-like” layer covering the intestinal epithelium and, therefore, preventing the adhesion of the pathogen or its toxins (Ruas-Madiedo et al., 2010; Fanning et al., 2012). Thus, to test the biofilm-formation ability of our EPS-producing bacteria different methodologies and abiotic surfaces were used as an initial approach before demonstrating this capability in biotic surfaces which, currently, is a challenging issue (**Figure 5**). The recently developed impedance-based method to monitor in real time the bacterial biofilm formation upon gold-microelectrodes of RTCA E-plates (Gutiérrez et al., 2016) showed that the ropy S89L strain presented a lower adhesion ability than the non-ropy DSM10140 parental one during the incubation period (**Figure 5A**). To discard any influence of the abiotic material on the adhesion ability, 24 h-old biofilms were formed upon glass-cover or polystyrene and compared with 46 h-old biofilms made of RTCA E-plates; all biofilms were stained with crystal violet and this method also supported the same tendency between both strains (*p* < 0.05) regardless of the abiotic surface considered (**Figure 5B**). However, statistical differences were noted when the behavior of each strain in the three abiotic surfaces was compared; for strain S89L the lowest biofilm formation capability was upon gold and polystyrene, whereas strain DSM10140 showed better adherence to gold (**Figure 5B**). This variability could be related to variations in the hydrophilic/hydrophobic nature of both bacterial surfaces that could suppose variable affinities for materials with different physical properties. In fact, it has been proved that the polar and non-polar characteristics of abiotic surfaces are involved in the differential capability to form biofilms of some pathogens (Meira et al., 2012).

**FIGURE 6:**
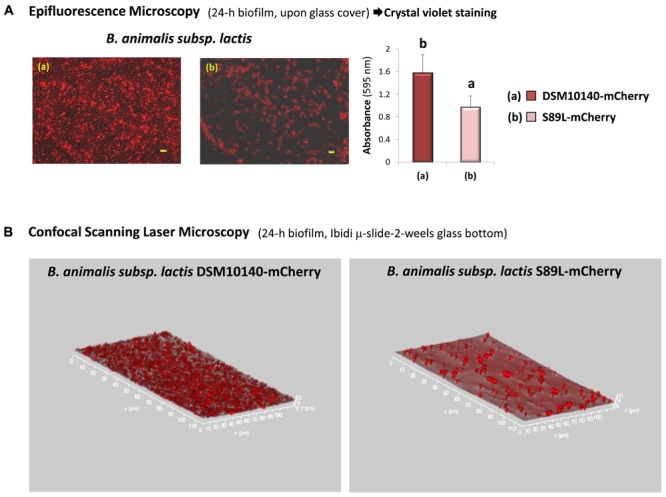
Detection of fluorescence-labeled bifidobacterial DSM10140-mCherry and S89L-mCherry strains upon glass covers using the epifluorescence microscope (bar 5 μm) and crystal violet quantification of the biofilms formed upon the glass covers; bars that do not share a common letter are significantly (*p* < 0.05) different **(A)**. Rotated 3D-images obtained from the Z- projection (thickness about 2–4 μm) showing the biofilm formed by *B. animalis* subsp. *lactis* DSM10140-mCherry upon glass μ-slides and the absence of biofilm by strain S89L-mCherry **(B)**.

The visualization by epifluorescence microscopy of 24 h-old biofilms formed with the fluorescent-labeled strains showed a higher bacterial density on the glass covered with the non-ropy DSM10140-mCherry strain, in comparison with the ropy S89L-mCherry ones (**Figure 6A**); a quantitative test performed with the same strains confirmed this difference (*p* < 0.05). Similarly, when intact bifidobacterial biofilms formed upon μ-slides were visualized with CSLM a very thin 3-D structure was detected only for strain DSM10140-mCherry whereas only residual bacteria remained attached for strain S89L-mCherry (**Figure 6B**). As far as we could note, this is the first report showing the biofilm formation by *Bifidobacterium* spp. and depending on the type of polymer that is synthesized it could favor or prevent the adhesion of the bifidobacteria to different abiotic surfaces. As it was indicated above, the presence of a HMW-EPS fraction could reduce the contact of other surface adhesins with the abiotic surface, thus avoiding the biofilm formation.

Therefore, all approaches used showed a reduced capability of the HMW-EPS-producing strains to form biofilms upon abiotic surfaces, as was also observed in the biotic (HT29) surface. Our observation confirms that previously reported with the high molecular mass EPS synthesized by *L. rhamnosus* GG (38) and *L. johnsonii* FI9785 (Dertli et al., 2015). The molecular weight of the HMW-EPS synthesized by these lactobacilli as well as our strain S89L was equal or higher than 1 × 10^6^ Da; whereas, the most abundant polymer fractions in the DSM10140 had lower molecular weight (less than 3 × 10^4^ Da). Therefore, although it cannot be discarded that some specific EPS could form a biofilm layer on the gut surface, other mechanisms could be involved in the capability of EPS-producing bacteria to counteract the adhesion of pathogens. In this regard, our previous observations with polymers purified from lactobacilli and bifidobacteria suggest that these polymers could also act as analogs of the eukaryotic PRR for pathogens, i.e., having a “lectin-like” activity, and therefore reducing their adhesion to the gut mucosa (Ruas-Madiedo et al., 2006). In any case, the contribution of EPS to counteracting microbial dysbiosis caused by infections deserves future research (Reid et al., 2011).

To sum up, our results show that fluorescent labeling of *B. animalis* subsp. *lactis* can be achieved by using green and mCherry fluorescent proteins but that these genes need to be under the control of a promoter from this species. It was demonstrated that the introduction of Balat-1410 mutation into the chromosome of parental DSM10140 strain was linked to the production of a larger abundance of a HMW-EPS, which in turn confers a ropy phenotype. Tagging bifidobacteria with mCherry allowed us to determine that some relevant functional characteristics of the strains, such as the adhesion to human intestinal cells or the capacity to form biofilms, are associated with the presence/absence of this ropy phenotype. The novel recombinant strains obtained in this work are a valuable tool to study the cross-talk mechanisms between bifidobacteria and host cells, and can make possible the development of further approaches to elucidate the role of these bacteria in complex *in vivo* systems. Furthermore, similar methodological approaches are required to demonstrate the functional properties of NGP bacteria which must be used to decipher the role of surface molecules involved in the beneficial properties attributable to beneficial microorganisms.

## Author Contributions

CH-C, PR-M, and AM contributed with the conception, experimental design and results interpretation of this study. NC-B carried out all experiments, some of them performed with the collaboration of MR-C. PR-M was in charge of the statistical analyses. AM and PR-M were in charge of writing the drafted manuscript. All authors performed a critical revision of the manuscript and approved the final version.

## Conflict of Interest Statement

The authors declare that the research was conducted in the absence of any commercial or financial relationships that could be construed as a potential conflict of interest.
