# Supplementary material for: Gene Replacement and Fluorescent Labeling to Study the Functional Role of Exopolysaccharides in Bifidobacterium animalis subsp. lactis
Source: Front Microbiol. 2017 Jul 25;8:1405. doi: 10.3389/fmicb.2017.01405 (PMC5524739; doi:10.3389/fmicb.2017.01405)

**Supplementary Figure S1**. Absence of auto-fluorescence, in the range of GFP (left panel) and mCherry proteins (right panel), in the non-labeled bifidobacterial strains (DSM10140 and S89L) using the Cytomics FC500 flow cytometer.


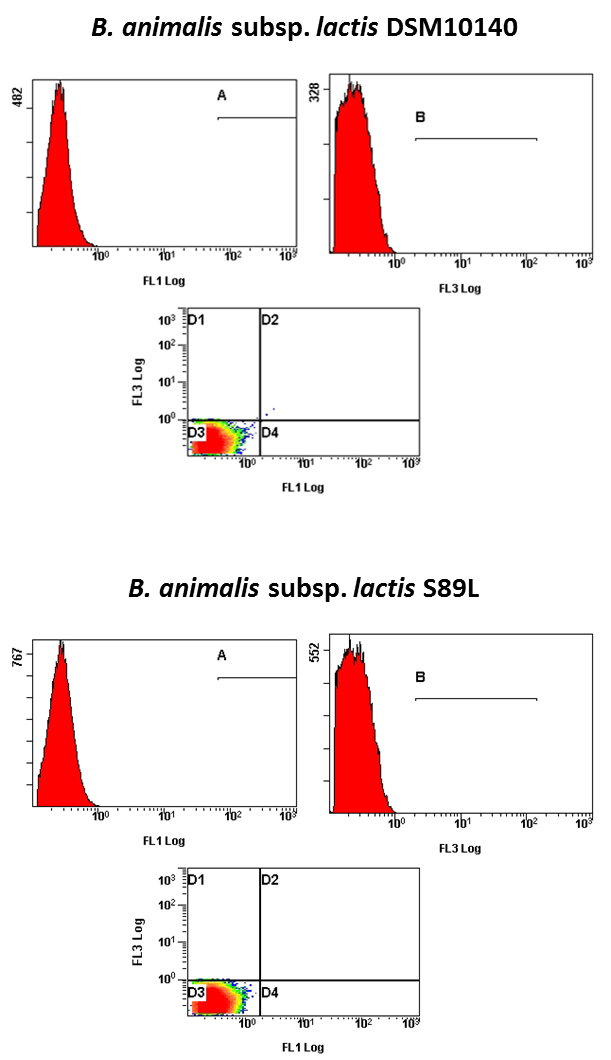

Supplement: Supplementary file 1 [file Data_Sheet_1.docx]
