# Supplementary material for: Gene Replacement and Fluorescent Labeling to Study the Functional Role of Exopolysaccharides in Bifidobacterium animalis subsp. lactis
Source: Front Microbiol. 2017 Jul 25;8:1405. doi: 10.3389/fmicb.2017.01405 (PMC5524739; doi:10.3389/fmicb.2017.01405)

**Supplementary Figure S2**. Data from a representative experiment used to obtain the linear regression equations between bacterial counts (CFU/ml) and “fluorescence emitted” (total number of events multiplied by the mean fluorescence intensity) detected by flow cytometry of serial dilutions made for the four *B. animalis* subsp. *lactis* strains harboring plasmids with fluorescent proteins. The coefficient of determination (R^2^) was included to show the fit of these data to the linear regression equations. Experiments were performed at least in triplicate.


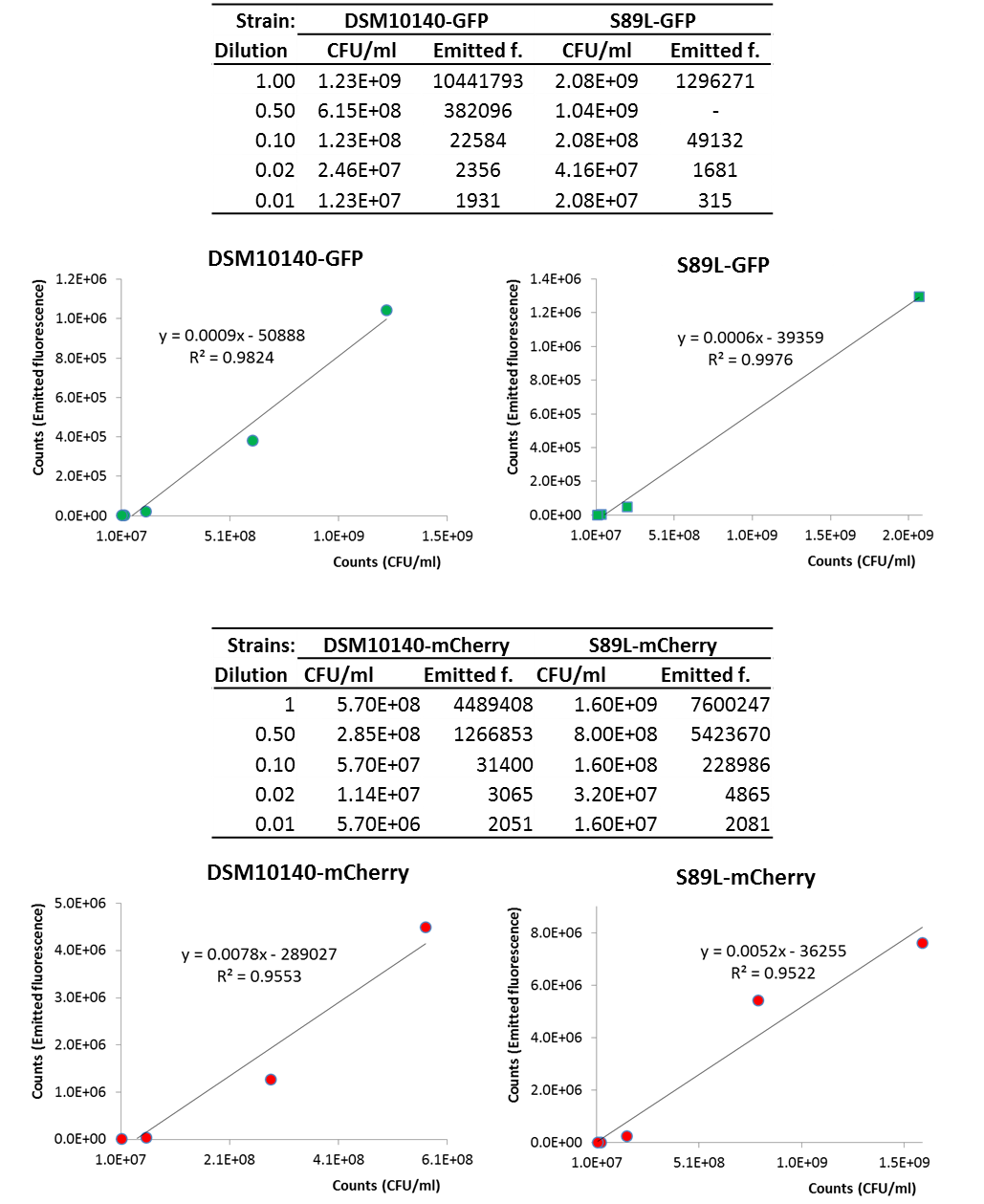

Supplement: Supplementary file 2 [file Data_Sheet_2.docx]
